# Resting and feeding preferences of *Anopheles stephensi* in an urban setting, perennial for malaria

**DOI:** 10.1186/s12936-017-1764-5

**Published:** 2017-03-10

**Authors:** Shalu Thomas, Sangamithra Ravishankaran, N. A. Johnson Amala Justin, Aswin Asokan, Manu Thomas Mathai, Neena Valecha, Jacqui Montgomery, Matthew B. Thomas, Alex Eapen

**Affiliations:** 1 IDVC Field Unit, National Institute of Malaria Research (ICMR), NIE Campus, 2nd Main Road, TNHB, Ayapakkam, Chennai, 600 077 India; 2 Department of Zoology, Madras Christian College, Tambaram, Chennai, 600 059 India; 3 National Institute of Malaria Research (ICMR), Sector 8, Dwarka, New Delhi, 110 077 India; 4 Department of Entomology, The Pennsylvania State University, University Park, PA 16802 USA

**Keywords:** Vector control, *Anopheles stephensi*, Urban malaria, Parity, Blood meal analysis

## Abstract

**Background:**

The Indian city of Chennai is endemic for malaria and the known local malaria vector is *Anopheles stephensi*. *Plasmodium vivax* is the predominant malaria parasite species, though *Plasmodium falciparum* is present at low levels. The urban ecotype of malaria prevails in Chennai with perennial transmission despite vector surveillance by the Urban Malaria Scheme (UMS) of the National Vector Borne Disease Control Programme (NVBDCP). Understanding the feeding and resting preferences, together with the transmission potential of adult vectors in the area is essential in effective planning and execution of improved vector control measures.

**Methods:**

A yearlong survey was carried out in cattle sheds and human dwellings to check the resting, feeding preferences and transmission potential of *An. stephensi*. The gonotrophic status, age structure, resting and host seeking preferences were studied. The infection rate in *An. stephensi* and *Anopheles subpictus* were analysed by circumsporozoite ELISA (CS-ELISA).

**Results:**

Adult vectors were found more frequently and at higher densities in cattle sheds than human dwellings. The overall Human Blood Index (HBI) was 0.009 indicating the vectors to be strongly zoophilic. Among the vectors collected from human dwellings, 94.2% were from thatched structures and the remaining 5.8% from tiled and asbestos structures. 57.75% of the dissected vectors were nulliparous whereas, 35.83% were monoparous and the rest 6.42% biparous. Sporozoite positivity rate was 0.55% (4/720) and 1.92% (1/52) for *An. stephensi* collected from cattle sheds and human dwellings, respectively. One adult *An. subpictus* (1/155) was also found to be infected with *P. falciparum*.

**Conclusions:**

Control of the adult vector populations can be successful only by understanding the resting and feeding preferences. The present study indicates that adult vectors predominantly feed on cattle and cattle sheds are the preferred resting place, possibly due to easy availability of blood meal source and lack of any insecticide or repellent pressure. Hence targeting these resting sites with cost effective, socially acceptable intervention tools, together with effective larval source management to reduce vector breeding, could provide an improved integrated vector management strategy to help drive down malaria transmission and assist in India’s plan to eliminate malaria by 2030.

## Background

Various factors like host preference, resting and feeding behaviour, adult longevity and density, human biting rate and host location strategy influence the role of mosquitoes in malaria transmission [1]. The state of Tamil Nadu had 8714 malaria cases in 2014, of which 337 (3.86%) were *Plasmodium falciparum* and the remaining 8377 (96.13%) *P. vivax* [2]. Almost 70% of the malaria cases recorded in Tamil Nadu occur in Chennai [3]. Malaria is endemic in Chennai, transmitted by the urban vector, *Anopheles stephensi,* which breeds predominantly in overhead tanks besides, other water storage habitats [4], and lesser examined rain fed clear water habitats. The transmission is unstable, but perennial with seasonal peaks mainly in July to August and then from October to November [5]. The present study was done as a part of the project on ‘Center for the Study of Complex Malaria in India’ (CSCMi) where transmission dynamics (micro environmental profile, immature and adult vector density, host, resting and breeding preferences of the vector mosquitoes) and eco-epidemiology of malaria (clinic study to investigate the impact of complex malaria on disease outcome in symptomatic individuals and community study to determine the incidence and prevalence rate of complex malaria including asymptomatic malaria) in urban transmission settings [6]. The main objective of this study was to find the feeding and resting preferences of *An. stephensi* and its transmission potential in a malaria endemic area of Chennai with perennial transmission.

## Methods

### Adult vector collections

**Fig. 1:**
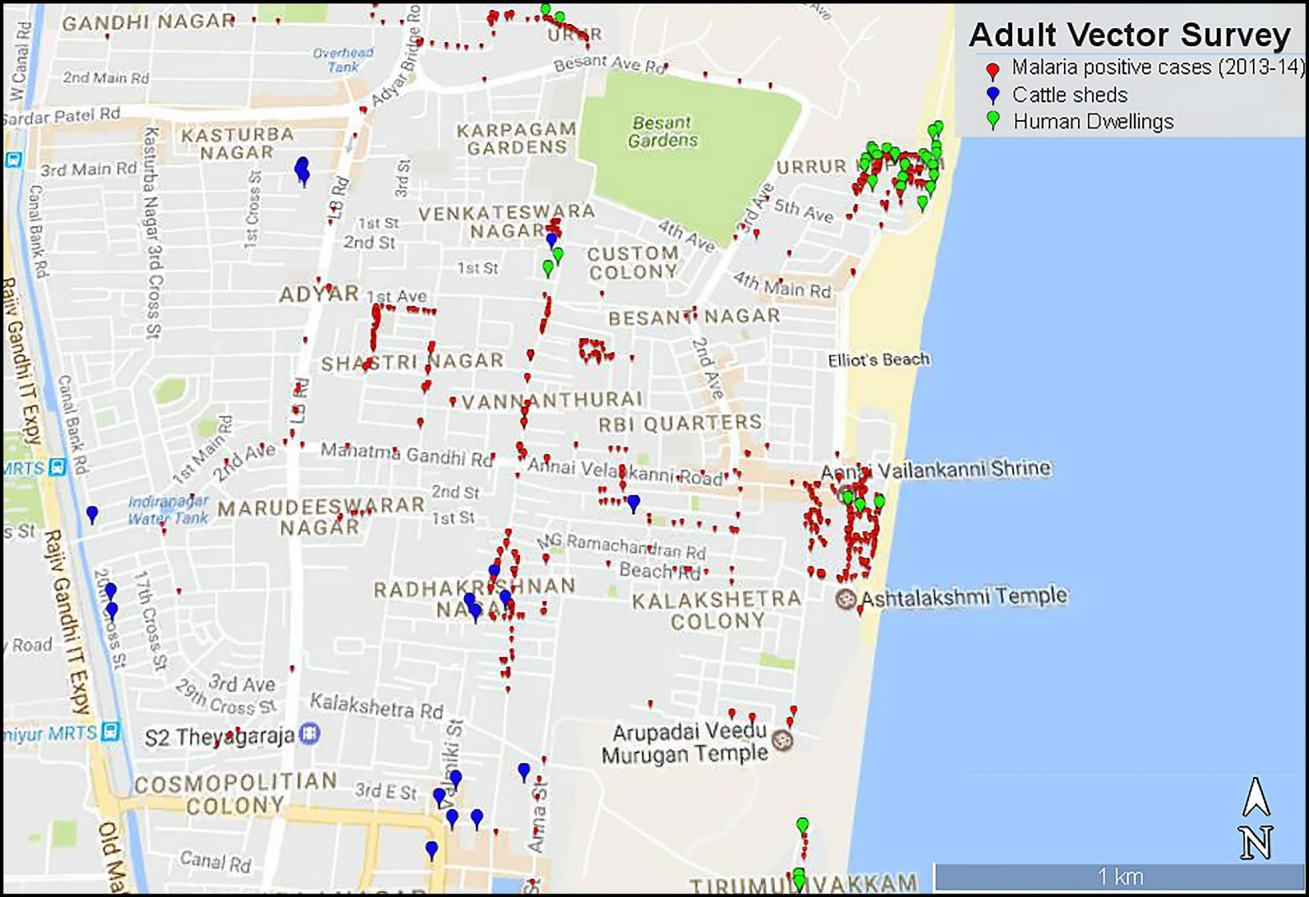
Study site with GPS plots of cattle sheds, human dwellings and malaria incidence of 2013–2014

**Fig. 2:**
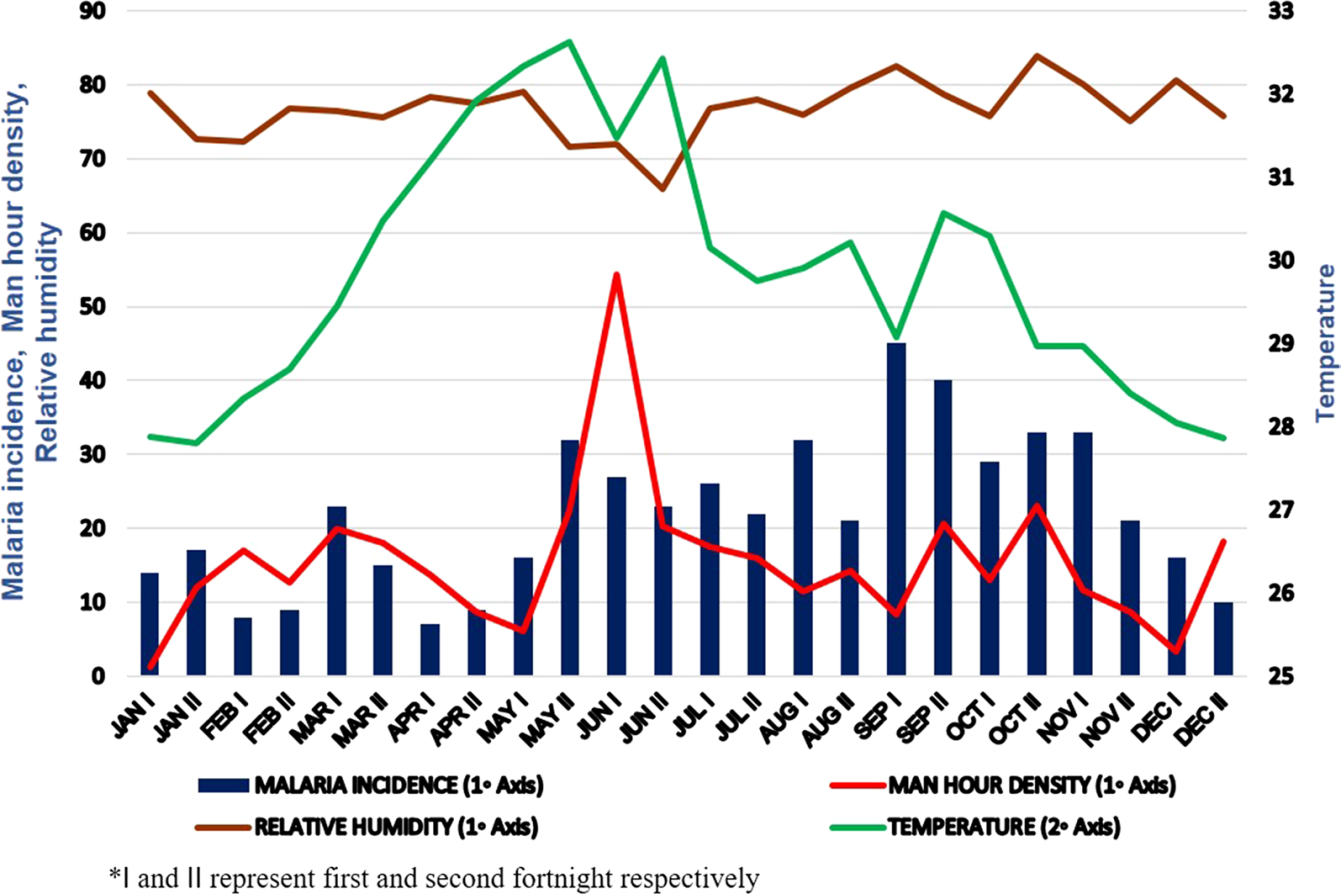
Man hour density of *Anopheles stephensi* with temperature, relative humidity and malaria incidence in Besant Nagar, Chennai for 2014

The study site, Besant Nagar (13.0002°N, 80.2668°E) is a residential area with slums adjacent to the seashore in the southeastern part of Chennai; it is distinctly characterized by its meso-endemic perennial transmission of malaria, predominantly *P. vivax*, by the Asiatic urban malaria vector, *An. stephensi*. Human dwellings (tiled, asbestos and thatched houses) and cattle sheds were surveyed from January to December 2014 in order to find the resting and feeding preferences besides, infectivity rate of the primary vector, *An. stephensi*. The study site indicating cattle sheds were georeferenced and human dwelling collection sites along with malaria incidence of 2014 is represented in Fig. 1. Cattle sheds were classified according to type of roof structures such as asbestos, thatched and concrete, visited on two consecutive days in a fortnight in the dusk during the study period using flashlight and a mouth aspirator (Table 1). A total of 17 cattle sheds were selected for yearlong survey. However, six were surveyed on regular basis (66.1% of total surveys), while other sheds were surveyed randomly (33.9% of total surveys) depending on the availability and accessibility to survey. In each cattle shed, 15–30 min were spent depending on the size/area of the cattle shed and presence or absence of mosquitoes at the time of collection. The fortnightly man hour density (MHD) is plotted against malaria incidence data of 2014, obtained from the Regional Office for Health and Family Welfare (ROH & FW), Besant Nagar, Chennai and corresponding temperature and relative humidity (using Onset HOBO data logger–U10-003) from a longitudinal temperature study carried out in parallel (Fig. 2).

**Table 1:** *Anopheles stephensi* collections from varied structure types of cattle sheds (by IRC) and human dwellings (by PSC)

Resting habitats	Roof types	No. of times surveyed	No. of times positive for resting habitats with *Anopheles stephensi* n (%)	No. of *Anopheles stephensi* collected	Abdominal conditions				
					Unfed	Freshly fed	Late stage fed	Semi gravid	Gravid
Cattle sheds (n = 17)^b^	Asbestos (n = 6)	79	46 (58.2)	474	34	30	371	24	15
	Concrete (n = 7)	46	14 (30.4)	75	3	3	68	1	0
	Thatched (n = 3)	38	14 (36.8)	96	2	5	66	20	3
	Roofless^a^ (n = 1)	20	10 (50.0)	98	3	7	61	22	5
Human dwellings (n = 245)^c^	Tiled (n = 6)	6	1 (16.7)	2	0	0	0	0	2
	Thatched (n = 188)	188	28 (14.9)	49	5	0	23	9	12
	Asbestos (n = 51)	51	1 (2.0)	1	1	0	0	0	0

^a^Open with concrete wall

^b^Sampling repeated

^c^One time survey

In addition, Pyrethrum spray sheet collections (PSC) were done in human dwellings with different roof types such as thatched, asbestos, and tiled, randomly selected in and around the malaria endemic area of about 3.5 km north–south and 2.5 km east–west direction [4]. They were surveyed on weekly basis to check the presence of resting adult vector mosquitoes, if any. Attempts were made to collect vector mosquitoes in concrete structures too. Since *Anopheles* mosquitoes were not observed, collections were focused on thatched, asbestos and tiled structures only. The collections were done during dawn (6.00–7.30 a.m.) in five to seven houses, spending 20–30 min for each house on every occasion. Indoor resting collections (IRC) were also performed with the help of mouth aspirator and flashlight, though it was not successful due to sparse sample count (Table 1). A few *Anopheles subpictus* specimens, caught during the fortnight collections were also screened to determine the presence of malaria parasites.

### Abdominal condition, age structure, host seeking preferences and infectivity

The collected mosquitoes were identified to the species level following standard identification keys [7, 8], females enumerated and graded based on their abdominal conditions. The late stage fed, gravid and/or semi-gravid appearance of the abdomen were considered as resting stages, while the unfed guts and/or freshly fed as feeding stages [9]. The fortnight man-hour density (MHD) of *An. stephensi* was calculated by dividing the total number of female mosquitoes collected by total time spent for a particular fortnight for 1 h period i.e., (Total female *An. stephensi* collected/Total time spent) ×60. Out of 634 (late stage fed and freshly fed mosquitoes together), 548 i.e., 86.4% (Table 1) were processed for blood meal analysis. After smearing the blood from the abdomen of female mosquitoes on Whatman filter paper (No. 1), the head and thorax of the mosquitoes were processed for circumsporozoite sandwich enzyme-linked immunosorbent assay (ELISA) following the Malaria Research and Reference Reagent Resource Center (MR4) protocol [10]. The dried blood spots were analysed by counter-current immunoelectrophoresis method [11]. A proportion of unfed and gravid females were immediately screened for the presence of sporozoite by dissecting the salivary glands and midguts for oocysts and the age structure or parity (gonotrophic status) of the vector population was ascertained by dissecting the ovaries and examining the tracheoles. All the dissections were done following the standard MR4 protocols [12]. The remaining unfed and gravid females were also processed for CS-ELISA.

## Results

The adult vectors (*An. stephensi*) were predominantly found resting in cattle sheds that were in close proximity (<5 metres distance and often with shared walls) to human dwellings. Fewer mosquitoes (both in terms of absolute numbers and frequency of structure types found positive) were collected from human dwellings. The adult vector density was relatively stable across the year, though with a maximum count observed during the first fortnight of June.

Mosquitoes were found in all types of cattle sheds (i.e., whether they were asbestos structures, concrete, thatched, or roofless sites with concrete walls). On the other hand, in 245 surveys of human dwellings, anopheline mosquitoes were rarely found and these tended to be encountered in thatched structures as opposed to tiled or asbestos structures. Among 882 *Anopheles* specimens collected from cattle sheds, 743 (84.2%) were *An. stephensi* (all female) and the remaining 139 (15.8%) were *An. subpictus* and *Anopheles vagus*. Out of 94 *Anopheles* species collected from human dwellings, 52 (55.3%) were *An. stephensi,* 41 (43.6%) *An. subpictus* and 1 (1.1%) *An. vagus.* 49 (94.23%) of the *An. stephensi* collected were from thatched structures followed by 2 (3.85%) from tiled structures and the remaining 1 (1.92%) from asbestos roofed house. When the abdominal conditions of the vector mosquitoes collected were examined, it was found that, among the mosquitoes collected from cattle sheds, 5.7% were unfed, 6% freshly fed, 76.2% of late-fed stage, 9% semi-gravid and the rest 3.1% gravid. Interestingly, 88.3% of the vectors found to be resting were late stage fed, semi gravid and gravid stages, while 11.7% were feeding stages (unfed and freshly fed). Similarly in human dwellings, 88.5% were late stage fed, semi gravid and gravid, while 11.5% were feeding stages. The freshly fed females were collected while they were feeding on cattle. The resting stage of vector mosquitoes were 7.5 times more than the feeding stages.

### Host-seeking preference of vector mosquitoes

Blood meal analysis was done using counter-current immunoelectrophoresis technique and the results are depicted in Table 2. Among the vector mosquitoes collected during 2014, 530 mosquitoes from cattle sheds and 18 from human dwellings were analysed for origin of their blood meal. Of 548 samples, 518 (94.5%) had bovine blood meal while only five were positive for human blood (four from human dwellings). Blood meal origin of 25 samples was unknown and the Human Blood Index (HBI) was 0.009. In contrast, Bovine Blood Index (BBI) was 0.95.

**Table 2:** Host seeking preference, *Plasmodium vivax* and *Plasmodium falciparum* infectivity of *Anopheles stephensi*

Resting habitats	Host seeking preference					Proportion of infectivity			
	No. of samples analysed	Bovine	Human	Mixed	Unknown	No. of samples analysed	*Pv* 210 n (%)	*Pv* 247 n (%)	*Pf* n (%)
Cattle sheds	530	518	1	0	11	720	3 (0.4)	0 (0.0)	1 (0.1)
Human dwellings	18	0	4	0	14	52	1 (1.9)	0 (0.0)	0 (0.0)
Total	548	518	5	0	25	772	4 (0.5)	0 (0.0)	1 (0.1)

### Age structure and malaria parasite infection in vector mosquitoes

About 200 females were dissected to check the presence of oocysts and sporozoites besides, age structure. None of them were found to harbor oocysts and sporozoite infections. Further, when the parity was checked, 57.75% of the dissected vectors were nulliparous, whereas, 35.83% were monoparous and 6.42% biparous. Cattle shed and household mosquito samples were processed for sporozoite detection by CS-ELISA and the results are shown in Table 2. A total of 772 samples collected during 2014 (720 from cattle sheds and 52 from households) were analysed for vector incrimination. Of these, five were found to be infected with the malaria parasites (four *P. vivax* 210 infected—three from cattle sheds and one from human dwelling) and one *P. falciparum* infected (collected from cattle shed). Sporozoite positivity rate was 0.648 and 1.92% of *An. stephensi* collected from human dwellings were infected. The *P*. *falciparum* infected mosquito sample was collected during February 2014 whereas, *P. vivax* infected samples were obtained during October, November and December. However, *P. vivax* infected sample from human dwelling was collected during March 2014. Furthermore, 135 *An. subpictus* samples collected during the study period were subjected to vector incrimination using CS-ELISA and one *P. falciparum* infection was detected.

## Discussion

In the present study, vectors were more abundant in outdoor structures (cattle sheds) than human dwellings. Wherever mosquitoes were found indoors, there appeared to be a preference for thatched structures, possibly due to the availability of eaves and crevices, which provide suitable/preferential hideouts and conducive humidity [13, 14]. Previous studies in Sri Lanka, Tanzania and Gambia, revealed poor housing similar to thatched structures to be associated with increased entry and resting of mosquitoes with high malaria incidences [13]. In addition, thatched structures mainly belong to households with low socio-economic status and these households tend to use fewer repellents against nuisance mosquitoes. Use of repellents has been associated with less malaria in a clinic study carried out in Chennai [15].

Abundance of vectors in cattle sheds confirms the previous finding of *An. stephensi* that, it prefers animal sheds for feeding besides, resting in Chennai [16–18]. It was observed that 96.77% of the cattle sheds were with shared walls (<5 metres distance) from the nearest human dwelling. The proximity of the cattle sheds to human dwellings indicates, even though the human host was so close, the mosquitoes perhaps were lured to cattle/animals for their blood meal as there was no repellent or any mosquito prevention method and therefore, the animals were easily bitten by vector mosquitoes. This was reflected in the blood meal analysis, which showed the vector mosquitoes preferring animal blood. The results of the blood meal analysis indicated the selective preference of vectors on animal blood as the mosquitoes preferred the cattle population, even though human hosts were abundant when compared to the former [19]. However, studies carried out in Iran reported endophagic and endophilic behaviour of *An. stephensi* [20].

Further, the human blood meal samples collected from cattle sheds, support the previous reports that, after feeding on human blood, *An. stephensi* used animal sheds for resting which would help in completing its gonotrophic cycle [17]. Four of the five infected mosquitoes were collected from cattle sheds/outdoors. In the present study, it is assumed that *An. stephensi* selected cattle sheds as foraging and as an ideal resting habitat. The density of resting adults was low, which is the same elsewhere in many urban cities/towns in India [4, 21, 22].

The sporozoite positivity rate observed was very low despite perennial transmission of malaria over a period of time. This might be due to the incomplete knowledge of resting preferences [23, 24], which means the current study probably underestimates the density of infectious mosquitoes. However, the relatively low infectivity rate in the mosquitoes could also be due to the fact that this area has a moderate to low transmission rate, or the infected mosquitoes undergo enhanced mortality [25–28]. The parity analysis supported the fact that, survival of the adult mosquitoes in the study area was limited. This was in line with studies carried out elsewhere that only 10% of the adult population survives to the epidemiologically relevant age  [29, 30]. In Solomon Islands, *P. falciparum* was detected from 15.2% of 1-parous *Anopheles farauti* mosquitoes, using PCR detection method [31]. Another study in Benin showed that, the infected females were at least of biparous stage of physiological age [32].

The results of ELISA (Table 2) showed that, *P. falciparum* infection was observed in mosquitoes collected during February, and *P. vivax* infection in mosquitoes collected during October, November and December. Being a malignant parasite, presence of *P. falciparum* in mosquitoes during relatively low transmission season (Fig. 2) underlines the importance of having intense active surveillance irrespective of seasons. However, with such low sample sizes it is difficult to draw any conclusions regarding seasonality or potential links to microclimate data [23].

*Anopheles subpictus* infected with malaria parasite is yet another interesting finding as this is the first report of vector incrimination of *An. subpictus* from Chennai. In India, sporozoite positive specimens were collected from a coastal village in the state of Tamil Nadu [33]. *Anopheles subpictus* was previously believed to be a benign species because of its zoophilic nature, though it was reported to be a vector in Sri Lanka and a secondary vector in Indonesia [34–39] and elsewhere, in Madhya Pradesh [40], Odisha [41] and urban area of Goa [42]. *P. falciparum* infection in *An. subpictus* indicates that it is a competent vector when favourable condition arises. Considering urban malaria, this finding is important as this can pose a real problem in future along with the primary/major vector, *An. stephensi* in Chennai.

### Limitations

The present study was centered on human dwellings and cattle sheds and the latter was found to be preferred resting place of the local vector. As the current understanding of the other resting preferences are obscure as far as the local vector is concerned, there are chances that the present estimates of vector density and sporozoite rate may underestimate the true picture in Chennai.

## Conclusion

The study revealed that adult vectors feed predominantly on cattle and also rest in cattle sheds. The UMS of the national programme focuses on larval source management, by carrying out anti-larval operations in breeding habitats of the urban malaria vectors. This strategy has additional benefits of contributing to Dengue and Chikungunya control as some of these habitats, like cisterns, are co-inhabited by *An. stephensi* and *Aedes aegypti* [4]. In rural areas, adult vector surveillance and control with indoor residual spray (IRS) or long lasting insecticidal nets (LLINs) often focus on indoor domestic dwellings. The present study indicated that human dwellings (other than thatched structures) are not the preferred resting sites of the urban malaria vector. Rather, mosquitoes appear to be most readily found resting in cattle sheds and feeding on cattle. A recent theoretical study identified that in areas where residual malaria transmission is sustained by zoophilic vectors, even modest amounts of control that explicitly target these vectors may dramatically reduce transmission [43]. Therefore, targeting these resting sites with appropriate, cost effective intervention tools that are socio-behaviourally acceptable, coupled with effective larval source management to reduce vector breeding, could create opportunities for improved integrated vector management strategies that will help drive down malaria transmission and assist in India’s plan to eliminate malaria by 2030.

## Abbreviations

CS-ELISA: circumsporozoite enzyme-linked immunosorbent assay
PSC: pyrethrum spray sheet collections
IRC: indoor resting collections
NVBDCP: National Vector Borne Disease Control Programme
UMS: Urban Malaria Scheme
IRS: indoor residual spray
HBI: Human Blood Index
BBI: Bovine Blood Index
MHD: man hour density

## References

[1] Sindato C, Kabula B, Mbilu TJNK, Manga C, Tungu P, Kazimoto JP (2011). Resting behaviour of *Anopheles gambiae* s.l. and its implication on malaria transmission in Uyui District, western Tanzania. Tanzan J Health Res..

[2] Malaria situation in India. http://nvbdcp.gov.in/Doc/mal_situation_Dec2014.pdf. Accessed 23 Dec 2015.

[3] Kumar DS, Andimuthu R, Rajan R, Venkatesan MS (2014). Spatial trend, environmental and socioeconomic factors associated with malaria prevalence in Chennai. Malar J..

[4] Thomas S, Ravishankaran S, Justin JA, Asokan A, Mathai MT, Valecha N (2016). Overhead tank is the potential breeding habitat of *Anopheles stephensi* in an urban transmission setting of Chennai, India. Malar J..

[5] National Vector Borne Disease Control Programme, Directorate General of Health Services, Ministry of Health & Family Welfare, Malaria Situation in India (State-wise) from 2009 to 2013. http://nvbdcp.gov.in/malaria11.html. Accessed 1 July 2014.

[6] Das A, Anvikar AR, Cator LJ, Dhiman RC, Eapen A, Mishra N (2012). Malaria in India: the center for the study of complex malaria in India. Acta Trop.

[7] Nagpal BN, Sharma VP (1995). Indian anophelines.

[8] Nagpal BN, Srivastava A, Saxena R, Ansari MA, Dash AP, Das SC (2005). Pictorial identification key for Indian anophelines.

[9] WHO. Manual on Practical Entomology in Malaria: Methods and techniques. Geneva: World Health Organization. Division of Malaria Other Parasitic Diseases, Part 2; 1975.

[10] Wirtz R, Avery M, Benedict M. Specific Anopheles techniques 3.3 *Plasmodium* Sporozoite ELISA. Malaria Research and Reference Reagent Resource Center, MR4 2007, p. 11.

[11] Nanda N, Bhatt RM, Sharma SN, Rana PK, Kar NP, Sharma A (2012). Prevalence and incrimination of *Anopheles fluviatilis* species S (Diptera: Culicidae) in a malaria endemic forest area of Chhattisgarh state, central India. Parasit Vectors..

[12] Benedict MQ. Methods in anopheles research. Malaria Research and Reference Reagent Resource Center (MR4) 2007.

[13] Atieli H, Menya D, Githeko A, Scott T (2009). House design modifications reduce indoor resting malaria vector densities in rice irrigation scheme area in western Kenya. Malar J..

[14] Bhattacharyya B, Bordoloi JP (2015). Effect of three different roofing materials on milk production of Jersey grade cows in different seasons: a field study in Guwahati. Res J Anim Vet Fish Sci..

[15] Van Eijk AM, Ramanathapuram L, Sutton PL, Peddy N, Choubey S, Mohanty S (2016). The use of mosquito repellents at three sites in India with declining malaria transmission: surveys in the community and clinic. Parasit Vectors..

[16] Vasanthi V. Field and laboratory studies on selected ecological and behavioral aspects of variants of *An. stephensi* Liston from south India. PhD thesis, Department of Zoology, University of Madras, India, 1996.

[17] Basseri H, Raeisi A, Khakha MR, Pakarai A, Abdolghafar H (2010). Seasonal abundance and host-feeding patterns of anopheline vectors in malaria endemic area of Iran. J Parasitol Res..

[18] Thapar BR, Sharma SN, Dasgupta RK, Kaul SM, Bali A, Chhabra K (1998). Blood meal identification by using Microdot ELISA in vector mosquitoes. J Commun Dis.

[19] Chaves LF, Harrington LC, Keogh CL, Nguyen AM, Kitron UD (2010). Blood feeding patterns of mosquitoes: random or structured?. Front Zool..

[20] Edalat H, Moosa-Kazemi SH, Abolghasemi E, Khairandish S (2015). Vectorial capacity and Age determination of *Anopheles stephensi* Liston (Diptera: Culicidae), during the malaria transmission in Southern Iran. J Entomol Zool Stud..

[21] Sahu SS, Gunasekaran K, Vanamail P, Jambulingam P (2011). Seasonal prevalence & resting behaviour of *Anopheles minimus* Theobald & *An. fluviatilis* James (Diptera: Culicidae) in east-central India. Indian J Med Res.

[22] Amala S, Aunradha V (2011). Diversity of mosquitoes in three foot hill villages of Sirumalai hills Dindigul, India. Arch Appl Sci Res..

[23] Cator LJ, Thomas S, Paaijmans KP, Ravishankaran S, Justin JA, Mathai MT (2013). Characterizing microclimate in urban malaria transmission settings: a case study from Chennai, India. Malar J..

[24] Sougoufara S, Diedhiou SM, Doucoure S, Diagne N, Sembene PM, Harry M (2014). Biting by *Anopheles funestus* in broad daylight after use of long-lasting insecticidal nets: a new challenge to malaria elimination. Malar J..

[25] Maier WA, Becker-Feldman H, Seitz HM (1987). Pathology of malaria-infected mosquitoes. Parasitol Today.

[26] Ferguson HM, Read AF (2002). Why is the effect of malaria parasites on mosquito survival still unresolved?. Trends Parasitol.

[27] Michel K, Kafatos FC (2005). Mosquito immunity against *Plasmodium*. Insect Biochem Molec Biol..

[28] Vernick KD, Oduol F, Lazzaro BP, Glazebrook J, Xu J (2005). Molecular genetics of mosquito resistance to malaria parasites. Curr Top Microbiol Immunol.

[29] Beck-Johnson LM, Nelson WA, Paaijmans KP, Read AF, Thomas MB, Bjornstad ON (2013). The effect of temperature on *Anopheles* mosquito population dynamics and the potential for malaria transmission. PLoS ONE.

[30] Zhao YO, Kurscheid S, Zhang Y, Liu L, Zhang L, Loeliger K (2012). Enhanced survival of *Plasmodium*-infected mosquitoes during starvation. PLoS ONE.

[31] Harada M, Ishikawa H, Matsuoka H, Ishii A, Suguri S (2000). Estimation of the sporozoite rate of malaria vectors using the polymerase chain reaction and a mathematical model. Acta Med Okayama.

[32] Anagonou R, Agossa F, Azondékon R, Agbogan M, Oké-Agbo F, Gnanguenon V (2015). Application of Polovodova’s method for the determination of physiological age and relationship between the level of parity and infectivity of *Plasmodium falciparum* in *Anopheles gambiae* s.s, south-eastern Benin. Parasit Vectors..

[33] Singh RK, Kumar G, Mittal PK, Dhiman RC (2014). Bionomics and vector potential of *Anopheles subpictus* as a malaria vector in India: an overview. Int J Mosq Res..

[34] WHO: ISBN: 9290222786. http://www.searo.who.int/entity/medicines/documents/9290222786/en/. April 2007.

[35] Jude PJ, Ramasamy R, Surendran SN (2014). Bionomic aspects of the *Anopheles subpictus* species complex in Sri Lanka. J Insect Sci..

[36] Chatterjee S, Chandra G (2000). Role of *Anopheles subpictus* as a primary vector of malaria in an area in India. J Trop Med Hyg..

[37] Surendran SN, Ramasamy R (2010). The *Anopheles culicifacies* and *An. subpictus* complexes in Sri Lanka and their implications for malaria control in the country. J Trop Med Hyg..

[38] Surendran SN, Sarma DK, Jude PJ, Kemppainen P, Kanthakumaran N, Gajapathy K (2013). Molecular characterization and identification of members of the *Anopheles subpictus* complex in Sri Lanka. Malar J..

[39] Sinka ME, Bangs MJ, Manguin S, Chareonviriyaphap T, Patil AP, Temperley WH (2011). The dominant *Anopheles* vectors of human malaria in the Asia-Pacific region: occurrence data, distribution maps and bionomic precis. Parasit Vectors..

[40] Kulkarni SM (1983). Detection of sporozoites in *Anopheles subpictus* in Baster district, Madhya Pradesh. Indian J Malariol.

[41] Kumari S, Das S, Mahapatra N (2013). *Anopheles subpictus* B and its role in transmission of malaria in Odisha, India. Trop Biomed.

[42] Kumar A, Hosmani R, Jadhav S, de Sousa T, Mohanty A, Naik M (2016). *Anopheles subpictus* carry human malaria parasites in an urban area of Western India and may facilitate perennial malaria transmission. Malar J..

[43] Waite JL, Swain S, Lynch PA, Sharma SK, Haque MA, Montgomery J (2017). Increasing the potential for malaria elimination by targeting zoophilic vectors. Sci Rep..

